# Intelligence Quotient in Patients with Myelomeningocele: A Review

**DOI:** 10.7759/cureus.3137

**Published:** 2018-08-13

**Authors:** Yusuf Alimi, Joe Iwanaga, Rod J Oskouian, Marios Loukas, R. Shane Tubbs

**Affiliations:** 1 Anatomy, St. George's University School of Medicine, St. George's, GRD; 2 Medical Education and Simulation, Seattle Science Foundation, Seattle, USA; 3 Neurosurgery, Swedish Neuroscience Institute, Seattle, USA; 4 Anatomical Sciences, St. George's University, St. George's, GRD; 5 Neurosurgery, Seattle Science Foundation, Seattle, USA

**Keywords:** myelomeningocele, spina bifida, hydrocephalus, intelligence quotient, venticuloperitoneal shunt

## Abstract

It has been proposed that hydrocephalus in children with myelomeningocele (MMC) can indicate a low intelligence quotient (IQ). Others have argued that it is not the mere presence of hydrocephalus but the superimposition of cerebrospinal fluid (CSF) infections, multiple shunt procedures, and other CNS complications that lead to the lowering of IQ in these patients.

In this paper, we review the literature to clarify the information about IQ in patients with MMC and whether it changes after infections and shunt procedures. We have also considered the other factors that could be involved in the IQ development of these patients and the differences revealed by the brain imaging of individuals with MMC.

The consensus remains that patients with MMC, with or without complications, tend to have a lower IQ than those without MMC. Hydrocephalus appears to decrease the IQ further in MMC patients. Some have proposed that prenatal repair of the MMC lesion reduces the need for ventricular shunting after birth, thus decreasing the risk of shunt complications such as a CNS infection, which can have a negative effect on IQ. More studies are needed to assess other risk factors (apart from folate deficiency) and genetic factors that could contribute to the development of MMC and their possible effects on patient IQ.

## Introduction and background

Myelomeningocele (MMC) is the most serious form of spina bifida. Its prevalence is about 1 per 1,000 births globally [1-2]. It has been proposed that MMC has a multifactorial pathogenesis, although its association with maternal folate deficiency is well known [1,3-4]. Although MMC is compatible with life, children with the condition often have a lower quality of life than normal individuals, mostly because of the central nervous system (CNS) impairment associated with this disorder [5].

Well-known abnormalities associated with the brain in individuals with MMC include hydrocephalus and the chiari II malformation, which is seen in almost all patients [1,3,5-8]. These abnormalities have been linked to various learning disabilities and to decreased executive functioning [3,5] (Meeting presentation: McLone DG. The effect of complications on intellectual function in 173 children with myelomeningocele. The International Society for Paediatric Neurosurgery, vii scientific meeting; September 16-19, 1979). Hydrocephalus has also been proposed as a significant predictor of intellectual capacity in MMC patients, and most studies of IQ and higher functioning in these individuals have focused on hydrocephalus with or without ventricular shunting [9-13]. It has also been proposed that complications secondary to ventricular shunting can worsen intellectual function [6].

## Review

Intelligence quotient and myelomeningocele

Intellectual capacity is an important measure by which an individual’s quality of life can be judged. Therefore, it is very important to know how the intelligence quotient (IQ) of children with MMC is affected by the death of neurons in utero [6,13]. There have been many studies of the effects of MMC on IQ in children [3,6,9,10,14-16]. They often demonstrate that the hydrocephalus can be a negative predictor of intellectual capacity in children with MMC, which is also detrimental to the quality of life in these patients [17]. Mapstone et al., found a significant difference in intellectual function when the IQs of MMC children with and without the hydrocephalus were compared [6]. Children with hydrocephalus had a low-normal IQ whereas those without hydrocephalus were in the normal range. Similar conclusions were drawn by Soare and Raimondi, who observed 173 MMC children, 133 of whom had developed hydrocephalus [13]. Their results showed that about 63% of the children with hydrocephalus had an IQ greater than 80, which in this study, was considered normal. However, 87% of the children without hydrocephalus had an IQ greater than 80. Children with hydrocephalus also scored notably lower than their siblings on tests assessing perceptual-motor function.

Soare and Raimondi also observed that individuals with a higher lesion level tended to have a lower IQ than their counterparts with a lower lesion level [13]. This was comparable to the findings of Mapstone et al., in that they observed as an inverse though statistically insignificant relationship between IQ and lesion levels [6]. However, Nejat et al. contradicted the above findings by showing no correlation between the level of the lesion and IQ [3]. Could this be because the sample size (50 children) in this latter study was much smaller than in the former (173), or were Mapstone et al. correct in deeming the relationship to be insignificant?

Several other studies have demonstrated a relationship between decreased cognitive function and hydrocephalus in individuals with MMC [10-11,13-14]. One major inclusion from the study by Hampton et al. was that although children with spina bifida performed more poorly in tasks involving spatial and executive function, their performance in vocabulary-related tasks was relatively good [14]. This might not mean much as children without MMC, who were used as controls, also performed much better on word-related than spatial tasks, so the findings could simply reflect a general trend.

It is notable that although executive functioning, learning, and memory-related tasks can be impaired in patients with MMC, the recognition of emotion is also deemed to be negatively affected, and some studies have argued that performance in tasks involving object-based visual processing could be intact in these patients [18-19]. Chiari II malformation has been proposed to have negative effects on performance IQ, verbal IQ, and visually-related tasks in patients with MMC [20].

Is hydrocephalus alone, when present in individuals with MMC, the sole predictor of IQ? Probably not. McLone showed that children with complicated hydrocephalus (i.e., hydrocephalus in combination with other CNS insults such as ventriculitis, meningitis, and an increase in shunt revisions) tended to have a significantly lower IQ than previously recorded intellectual function scores (meeting presentation: McLone DG, 1979, September 16-19). However, children with hydrocephalus (± shunt) but without complications had scores similar to their previous record. Other studies have yielded similar results, associating more frequent shunt revisions and complications with poorer functional outcomes [21-22]. McLone also observed a slight correlation between lesion location and infection rate such that children with a higher lesion had more infections and therefore a lower IQ (meeting presentation: McLone DG, 1979, September 15-18).

Lower socioeconomic status (SES) has generally been assumed to be concurrent with lower IQ [15,23]. It is therefore reasonable to inquire about the IQ of children with MMC born into low SES families. Swartwout et al. attempted to discern the relationship between MMC, IQ, and SES [24]. Although they did not look for a difference in IQ between children with and without MMC with low SES as the common variable, their results demonstrated that a lower SES in children with MMC is associated with a lower “verbal” IQ, which in turn tends to lead to a lower IQ [24].

Cortical organization and imaging of the brain in MMC

IQ is a measure of cortical function, and visualizing the differences in cortical organization and overall brain topography in patients with MMC can help to elucidate this disorder. It has been proposed that the cerebral cortex in individuals with MMC has an atypical organization leading to derangements in motor and cognitive functions [25]. Although heritability is prominent in IQ development, research has shown that people with higher IQ tend to have a prolonged period of cortical thickening during childhood [26]. But how does an atypical arrangement of the cerebral cortex lead to decreased cognitive function, what physical characteristics are seen in the cerebral cortex of these individuals, and how do these relate to IQ? To investigate these questions, Treble et al. studied the relationship between cortical thickness, IQ, and fine motor function in MMC [25]. Their position was that since many neurocognitive disorders are associated with atypical brain volume, it would be useful to know how the unusual cortical organization in individuals with MMC relates to their cortical thickness. The relationship between cortical thickness and IQ in individuals with MMC was studied, along with the question of whether there is an upper or lower limit to cortical thickness and gyrification for ideal motor and cognitive function in these individuals [25]. High-resolution magnetic resonance images (MRIs) of the brain were obtained from 64 patients with MMC and compared with 24 normally-developing controls, with each individual undergoing IQ and fine motor dexterity tests. Their results were matched for their respective age groups.

Treble et al. observed a negative correlation between IQ and increased cortical thickness in individuals with MMC and suggested that the greater the increase in cortical thickness the lower the IQ, resulting in poorer fine-motor and cognitive functions. At the other end of the spectrum, a low cerebral cortical thickness was associated with higher IQ. Therefore, the greater the deviation of cortical thickness from the norm, the lower the IQ [25].

Another imaging study showed a remarkable decrease in white matter and a corresponding increase in cerebrospinal fluid (CSF) in the brains of children with MMC [27]. These results suggested a decrease in myelination and disruption of white matter pathways secondary to hydrocephalus. This supports the consensus that hydrocephalus is associated with a greater IQ deficit in patients with MMC plus hydrocephalus than in those with MMC but no hydrocephalus. The negative influence of hydrocephalus on brain development in both humans and animals, with effects such as neuronal disruption and cellular death, is well documented, so it is of no surprise that IQ deficits are more pronounced in individuals with MMC and concomitant hydrocephalus [28]. Lindquist et al. found further evidence for the detrimental effect of hydrocephalus on learning and executive functioning: there was no significant difference in these cortical processes between children with MMC and hydrocephalus, and children with only hydrocephalus, with both having similarly low scores on tests for executive function compared to normal children [29].

Treatment of hydrocephalus in MMC and how it affects IQ

Since the consensus appears to be that children with MMC tend to have a sub-average IQ, and hydrocephalus in patients with MMC carries a negative IQ prognosis, it is important for us to know how, or if, these children can improve after treatment. The MMC lesion can be repaired by various surgical techniques [30]. Surgical treatment for MMC has declined during the past two decades owing to public awareness about folate supplementation before and during pregnancy [31]. There is an ongoing argument in the literature regarding prenatal versus postnatal repair of the lesion, with the former being associated with a lower incidence of hydrocephalus [32-34]. Hammock et al. were among the first groups to study the development of intellectual performance and IQ after surgery [10]. All eight of their patients, ranging from 30 months to 13 years and five months of age, had ventriculomegaly along with the MMC lesion but no apparent symptoms or signs of raised intracranial pressure. The patients underwent psychological testing along with cranial computed tomography (CT) preoperatively and their intraventricular pressures were continuously measured. Each underwent ventricular shunting and then psychological testing postoperatively [10]. Hammock et al. observed that over a 1-3 month period, there was a reversal and stabilization in the downward trend of intellectual performance, with a noticeably improved IQ performance in one patient within six months. Over a nine-month period, all but one of the children had significantly improved IQ scores [10].

Mapstone et al. performed a more standardized test in which the patients were divided into three groups [6]: Group I included children who never required shunting, Group II were children with shunting but with no CNS complications (e.g., ventriculitis), and Group III were children requiring shunting but with CNS complications. After CSF shunting, the average IQ of the children who never required shunting (Group I) was much higher than in the other two groups. Children in Group II also had a higher IQ average than those in group III [6]. The authors concluded that the downward trend in the IQ of Group III patients could be attributed to shunting complications or infections. Does this mean that if shunting complications or infections are minimized, these children would have a similar IQ to their counterparts in Group II? Interestingly, this was observed by Arrington et al., who showed that repeat shunt revisions can be associated with reduced cognition [9].

The results from most studies have shown that IQ loss in children with MMC is an inborn error that occurs early during embryogenesis, so no amount of treatment or correction can normalize the IQ [22,35]. However, if hydrocephalus is corrected early, further worsening of the patient’s IQ functioning can be prevented and the IQ returned to baseline for MMC patients [22]. 

## Conclusions

The debate about the causes of low IQ in many patients with MMC persists. Some have proposed that the prenatal repair of the MMC lesion reduces the need for ventricular shunting after birth and thereby decreases the risk of shunt complications and CNS infections, which normally have a negative prognosis for IQ. The consensus remains that patients with MMC, with or without complications, tend to have a lower IQ than those without the lesion. Hydrocephalus appears to decrease the IQ further in patients with MMC, although some studies have shown that if it is corrected in early age, the IQ can return to MMC baseline though not to normal levels. CNS infections also probably affect IQ negatively in these patients. More studies are needed to determine what other risk factors, apart from folate deficiency and genetic factors, can affect IQ in these patients.
